# Relationship between resistance training and lipoprotein profiles in sedentary male smokers

**Published:** 2008

**Authors:** Ina Shaw, Brandon S Shaw

**Affiliations:** Vaal University of Technology, Department of Marketing and Sport Management, Vanderbijlpark; Tshwane University of Technology, Department of Sport, Rehabilitation and Dental Sciences, Pretoria

## Abstract

Epidemiological studies have found plasma lipid and lipoprotein levels to be predictive of cardiovascular disease in adults. To date, regular aerobic modes of exercise have been associated with favourable alterations in lipid and lipoprotein levels. However, the effect of resistance training on lipid and lipoprotein levels is inconclusive and conflicting. Therefore, the aim of this study was to provide some clarity on whether resistance training could be used to improve sedentary male smokers’ lipoprotein profiles.

The study made use of a pre-test, a treatment period and a post-test. Subjects were placed into one of two groups, namely, a resistance-training (RES) group (*n* = 13) or a control (CON) group (*n* = 12). Throughout the 16-week experimental period the CON group received no treatment whatsoever. After resistance training, serum triglyceride levels were significantly decreased by 18.42% from 1.162 mmol/l (± 0.476) to 0.831 mmol/l (± 0.058) (*p* = 0.038) in the RES group. However, resistance training was found to have no impact on any of the other measured lipid and lipoprotein measures.

In conclusion, these findings indicate that resistance training appears to have no significant effect on lipid and lipoprotein profiles in sedentary male smokers and therefore cannot prevent the advance of CAD.

## Summary

Atherosclerosis is the leading cause of coronary artery disease(CAD) in most Western populations and is associated with an accumulation of cholesterol in the muscular walls of arteries.^1^ This finding is substantiated by the World Health Organisation which indicated that nearly half of the variance in CAD rates is due to differences in blood cholesterol levels.^2^ In South Africa, 80% of ‘westernised’ South Africans have an elevated cholesterol level, with the other 20% having cholesterol levels that place them at risk of developing hypercholesterolaemia.^3^

The terms ‘hyperlipidaemia’ and/or ‘hypercholesterolaemia’ should be considered misnomers since an increase in highdensity lipoprotein cholesterol (HDL-C) (hyperlipidaemia) must be seen as a negative risk factor and so the term dyslipidaemia should rather be utilised to describe this risk factor for CAD. Dyslipidaemia is generally characterised by hypertriglyceridaemia, an increased low-density lipoprotein cholesterol (LDL-C) and a decreased HDL-C.^4^

Exercise training plays an important role in improving lipid and lipoprotein levels, since dietary or caloric restriction alone has been shown to be an ineffective method of improving lipid and lipoprotein profiles in the long term.^5^-^7^ Exercise provides an additional benefit in that it decreases appetite in men (but not in women), effectively reducing caloric and lipid intake and accumulation of fat mass, which all result in favourable lipid and lipoprotein profiles.^8^ Conversely, intensive exercise training in the presence of high serum lipid levels may even aggravate the development of atheromatosis by releasing catecholamines which damage the vascular walls, leading to their susceptibility to lipid deposition.^9^

To date, regular aerobic modes of exercise have been associated with favourable alterations in lipid and lipoprotein levels.^10^-^12^ However, few studies have generated conclusive data on the effects of resistance training on lipid and lipoprotein levels. The effects of this mode of training are as yet not well documented and when such data are forthcoming, the findings are not consistent.^6^,^13^

Resistance training could be beneficial in improving lipid and lipoprotein levels since it results in an increased muscle lipoprotein lipase (LPL) and disparate decrease in hepatic LPL, which might result in favourable lipid and lipoprotein alterations. 14 The increase in muscle LPL and the decrease in hepatic LPL following resistance training may result in increased very low-density lipoprotein cholesterol catabolism and decreased HDL-C breakdown, respectively.^14^

The aim of this study was therefore to investigate whether resistance training could improve a sedentary male smoker’s lipid and lipoprotein profile.

## Methods

The study made use of a quantitative, pre-test/post-test research design. Twenty-five apparently healthy, sedentary male smokers between the ages of 20 and 35 years (mean age was 28 years and 7 months) volunteered to participate in the study. The subjects were informed about the potential risk of participation and gave their signed consent. Subjects were placed in either a control group (CON) (*n* = 12) or resistance-training group (RES) (*n* = 13). Sedentary male smokers were selected as the target sample group since cigarette smoking and physical inactivity are two of the major CAD risk factors and both place the sedentary smoker at risk for developing an unfavourable lipid and lipoprotein profile.

Each subject’s total cholesterol (TC), triglyceride (TG), LDL-C and HDL-C concentrations and TC:HDL-C, LDL-C: HDL-C and non-HDL-C (n-HDL-C) ratios/indexes were measured and/or calculated^6^,^15^-^17^ following a nine- to 12-hour fasting period and prior to any exercise.^6^,^17^

The exercise intensity for each subject in the RES group was calculated subsequent to each subject undergoing a 10-repetition maximum evaluation for each prescribed exercise. The results of each subject were recorded in terms of the weight lifted and the amount of repetitions, which had to be less than 10, to reach fatigue. Each subject’s one-repetition maximum (1-RM) was then calculated according to the following formula: 1-RM = (weight lifted) ÷ [1.0278 − (repetitions to fatigue × 0.0278)].^18^ The number of crunches prescribed to each subject was determined by the maximum number of repetitions that a subject could perform in 60 seconds.

Members of the CON group were instructed to maintain their usual activities and not to take part in any form of structured exercise during the experimental period. As with the experimental group, they were also not given any advice on their diet, alcohol intake and smoking behaviour over the experimental period.

The RES group subjects were instructed to exercise three times weekly for a period of 16 weeks. All exercise sessions were preceded by five minutes of easy cycling at a heart rate of less than 100 beats per minute (bpm), followed by eight stretching exercises performed for two sets of 30 seconds. Exercise sessions were concluded with five minutes of easy cycling at a heart rate of less than 100 bpm.^6^,^13^ Each RES group subject performed three sets of 15 repetitions using shoulder press, latissimus dorsi pull-downs, seated chest press, seated rows, crunches, unilateral leg press, unilateral knee extensions and unilateral prone leg curls at an intensity of 60% 1-RM.^13^ The subjects were allowed a 60- to 90-second rest period between each set.^19^ For crunches, each subject was required to perform three sets of 60% of the maximum number of repetitions that he had performed during the initial evaluation. Each individual’s 1-RM was re-evaluated every four weeks and his exercise programme adjusted accordingly.^13^

Descriptive statistics and dependant t-tests were calculated by the Rand Afrikaans University’s Statistical Consultation Service (STATKON) using the Statistical Package for Social Sciences (SPSS) II. A 95% confidence level (*p* ≤ 0.05) was used for determining a statistically significant change from the pre-test to the post-test.

## Results

With regard to the CON group, a non-significant 0.350% decrease in TC levels was found at the conclusion of the 16-week experimental period (*p* = 1.000) (Table 1). Even though the CON group had a 2.300% increase in LDL-C levels, this change was found to be non-significant (*p* = 0.754). Similarly, the increase in HDL-C levels was found to be non-significant (*p* = 0.754). With regard to the calculated indexes, the n-HDL-C and TC:HDL-C ratio in the CON group decreased non-significantly by 0.780% (*p* = 0.530) and 0.260% (*p* = 0.638), respectively. The CON group’s LDL-C:HDL-C ratio increased non-significantly by 2.870% from the pre- to the post-test (*p* = 0.875). The CON group’s TG levels increased significantly (albeit unfavourably) by 9.480% from 1.621 mmol/l to 1.761 mmol/l (*p* = 0.026).

**Table 1:** Pre- And Post-Test Lipoprotein Lipid Responses To 16 Weeks Of Resistance Training

*Group*	*Pre-test (SD)*	*Post-test (SD)*	*Difference (SD)*	*% change (significance)*
Total cholesterol (TC) (mmol/l)				
CON	5.062 (± 0.272)	5.043 (± 0.310)	0.019 (± 0.198)	0.350% decrease (*p* = 1.000)
RES	3.614 (± 0.819)	3.625 (± 0.666)	0.011 (± 0.477)	1.570% increase (*p* = 0.807)
Low-density lipoprotein cholesterol (LDL-C) (mmol/l)				
CON	4.121 (± 0.274)	4.209 (± 0.415)	0.088 (± 0.386)	2.300% increase (*p* = 0.754)
RES	2.859 (± 0.914)	2.621 (± 0.765)	0.238 (± 0.486)	7.000% decrease (*p* = 0.116)
High-density lipoprotein cholesterol (HDL-C) (mmol/l)				
CON	1.276 (± 0.165)	1.292 (± 0.231)	0.016 (± 0.170)	1.350% increase (*p* = 0.754)
RES	1.052 (± 0.264)	1.169 (± 0.299)	0.117 (± 0.211)	12.230% increase (*p* = 0.069)
Non-high-density lipoprotein cholesterol (n-HDL-C)				
CON	3.786 (± 0.288)	3.751 (± 0.286)	0.035 (± 0.211)	0.211) 0.780% decrease (*p* = 0.530)
RES	2.625 (± 0.865)	2.456 (± 0.767)	0.169 (± 0.433)	5.640% decrease (*p* = 0.152)
Total cholesterol: high-density lipoprotein cholesterol ratio (TC:HDL-C ratio)				
CON	4.031 (± 0.582)	4.003 (± 0.637)	0.028 (± 0.494)	0.260% decrease (*p* = 0.638)
RES	3.689 (± 1.242)	3.323 (± 1.105)	8.500% decrease (*p* = 0.173)
Low-density lipoprotein cholesterol:high-density lipoprotein cholesterol ratio (LDL-C:HDL-C ratio)				
CON	3.297 (± 0.599)	3.359 (± 0.683)	0.063 (± 0.596)	2.870% increase (*p* = 0.875)
RES	2.932 (± 1.340)	2.472 (± 1.140)	0.460 (± 0.912)	13.420% decrease (*p* = 0.152)
Triglycerides (TG) (mmol/l)				
CON	1.621 (± 0.377)	1.761 (± 0.391)	0.140 (± 0.201)	9.480% increase (*p* = 0.026*)
RES	1.162 (± 0.476)	0.831 (± 0.058)	0.332 (± 0.494)	18.420% decrease (*p* = 0.038*)

RES: resistance training group; CON: control group; SD: standard deviation, *significant.

Similar to the CON group, no significant change in mean TC concentration was observed following 16 weeks of RES training, with only a 1.570% increase being found at the post-test (*p* = 0.807). Despite the RES group demonstrating a 7.000% increase in LDL-C levels, this change was found to be non-significant (*p* = 0.116). Similarly, the increase in HDL-C levels was found to be non-significant following RES training (from 1.052 mmol/l to 1.169 mmol/l) (*p* = 0.069) as was the 5.640% decrease in n-HDL-C levels (from 2.625 to 2.456) (*p* = 0.152). Although the RES group was found to have an 8.500% reduction in the TC:HDL-C ratio, this reduction was found to be non-significant (*p* = 0.173). Moreover, the 16 weeks of RES training resulted in a large mean (albeit non-significant) 13.420% decrease in their LDL-C:HDL-C ratio (*p* = 0.152). In contrast, the RES training did result in a significant 18.420% decrease in TG levels from 1.162 mmol/l to 0.831 mmol/l (*p* = 0.038).

## Discussion

There is considerable consensus from existing literature and the findings of the present study that resistance training has no effect on TC levels.^6^,^13^,^15^,^17^,^20^,^21^ The present study’s finding that resistance training had no effect on LDL-C levels is also consistent with several studies.^6^,^13^,^16^,^17^,^20^,^22^,^23^ However, these findings are not unequivocal since several other studies have demonstrated that resistance training may indeed decrease LDL-C levels following a period of resistance training.^7^,^14^,^15^,^22^,^24^,^25^-^27^ What is perplexing is that there is no evident difference in study design (i.e. in terms of samples and exercise variables) between those studies that did not significantly alter LDL-C levels, implying that there are no definite variables ensuring success in lowering LDL-C levels.

The findings of several studies, including those of the present investigation, have found that resistance training had no effect on HDL-C levels,^7^,^13^,^16^,^17^,^20^-^23^,^26^ whereas in some instances, resistance training has been found to increase HDL-C levels.^6^,^14^,^15^,^22^,^25^,^27^ A possible explanation for this is that the studies that found increases in HDL-C made use of samples with lower baseline levels of HDL-C and shorter study periods.

Even though the determination of concentrations of small, dense LDLs, either by direct laboratory methods or via the calculation of n-HDL-C (TC minus HDL-C), is essential in determining CAD risk, few studies have focused on the effect of resistance training on this risk factor.^10^ There are almost no other studies therefore to which these findings can be compared.

The present study and other studies^13^,^17^ found that resistance training had no effect on the TC:HDL-C ratio. Most previous studies, however, have found that resistance training decreased this ratio,^6^,^7^,^15^,^22^,^26^ especially in men.^6^,^26^ As in the case of the present study, several studies have demonstrated a decrease in TG levels.^7^,^13^,^14^,^22^ However, resistance training has also been shown to have no effect^20^ or to unfavourably increase TG levels.^17^,^28^

The findings of the present study demonstrated that resistance training was not associated with favourable changes in lipid and lipoprotein levels in sedentary male smokers. Also, the sheer diversity of the different methods of applying resistance training is in itself a source of complication when attempting to determine if resistance training can indeed favourably alter lipid and lipoprotein profiles. Therefore, an optimal combination of resistance-training workload, intensity and number of repetitions still eludes researchers in this field. The contradictory findings in the literature, including the present findings, suggest that such an ‘optimal combination’ probably does not exist. It is therefore difficult to make a case for the use of resistance training to favourably alter the lipid and lipoprotein profiles of sedentary male smokers, and thus to reduce their risk of CAD.
